# Identification of Targets of a New Nutritional Mixture for Osteoarthritis Management Composed by Curcuminoids Extract, Hydrolyzed Collagen and Green Tea Extract

**DOI:** 10.1371/journal.pone.0156902

**Published:** 2016-06-08

**Authors:** Fanny Comblain, Jean-Emile Dubuc, Cécile Lambert, Christelle Sanchez, Isabelle Lesponne, Samuel Serisier, Yves Henrotin

**Affiliations:** 1 Bone and Cartilage Research Unit, Arthropôle Liège, University of Liège, CHU Sart-Tilman, Liège, Belgium; 2 Orthopedic Department, Cliniques Universitaires Saint-Luc, Brussels, Belgium; 3 Royal Canin Research Center, Aimargues, France; 4 Physical Therapy and Rehabilitation Department, Princess Paola Hospital, Vivalia, Marche-en-Famenne, Belgium; University of Umea, SWEDEN

## Abstract

**Objective:**

We have previously demonstrated that a mixture of curcuminoids extract, hydrolyzed collagen and green tea extract (COT) inhibited inflammatory and catabolic mediator’s synthesis by osteoarthritic human chondrocytes. The objective of this study was to identify new targets of COT using genomic and proteomic approaches.

**Design:**

Cartilage specimens were obtained from 12 patients with knee osteoarthritis. Primary human chondrocytes were cultured in monolayer until confluence and then incubated for 24 or 48 hours in the absence or in the presence of human interleukin(IL)-1β (10^-11^M) and with or without COT, each compound at the concentration of 4 μg/ml. Microarray gene expression profiling between control, COT, IL-1β and COT IL-1β conditions was performed. Immunoassays were used to confirm the effect of COT at the protein level.

**Results:**

More than 4000 genes were differentially expressed between conditions. The key regulated pathways were related to inflammation, cartilage metabolism and angiogenesis. The IL-1β stimulated chemokine ligand 6, matrix metalloproteinase-13, bone morphogenetic protein-2 and stanniocalcin1 gene expressions and protein productions were down-regulated by COT. COT significantly decreased stanniocalcin1 production in basal condition. Serpin E1 gene expression and protein production were down-regulated by IL-1β. COT reversed the inhibitory effect of IL-1β. Serpin E1 gene expression was up-regulated by COT in control condition.

**Conclusion:**

The COT mixture has beneficial effect on osteoarthritis physiopathology by regulating the synthesis of key catabolic, inflammatory and angiogenesis factors. These findings give a scientific rationale for the use of these natural ingredients in the management of osteoarthritis.

## Introduction

Osteoarthritis (OA) is a chronic, painful and inflammatory musculoskeletal disease. It affects the joints and generates functional impairment. OA is the most common joint disease and is associated with an abnormal remodeling of joint tissues. One major attribute of OA is the progressive degeneration of articular cartilage. Chondrocytes play a major role in cartilage degradation in OA by producing catabolic and inflammatory mediators and free radicals in response to mechanical or biochemical stimuli [1, 2]. These mediators are involved in cartilage extracellular matrix degradation. They also interact with synoviocytes and subchondral bone cells.

Nowadays, OA curative treatments are lacking. The goal of treatment in OA is to reduce pain and improve function. There is no cure for the disease, but some attempts to slow the progression of the disease. Current recommendations for the management of OA combine non-pharmacological and pharmacological interventions. Moreover, for patients suffering from severe OA, joint replacement is suggested [3]. Between non-pharmacological modalities, exercise, biomechanical interventions, weight loss if overweight or obesity, and thermal modalities are widely recommended [3–5]. Acetaminophen and non-steroidal anti-inflammatory drugs (NSAIDs) (topical or oral) are the most prescribed pharmacological treatments. Intra-articular corticosteroids are sometimes suggested for hip and knee OA [3–5]. However, NSAIDs and acetaminophen, when long-term used, may be associated with adverse effects, especially gastrointestinal detrimental effects [6].

Therefore, safer alternative interventions are needed. Such interventions could come from nutraceuticals [7–11]. We have previously demonstrated that a mixture of curcuminoids extract, hydrolyzed collagen and green tea extract inhibited inflammatory and catabolic mediator’s synthesis by human OA chondrocytes in monolayer [12]. The mixture was called COT. C was used for Curcuminoids extract, O for Hydrolyzed cOllagen and T for green Tea extract. More particularly, COT had an additive inhibitory effect on matrix metalloproteinase (MMP)-3 and IL-1β stimulated NO production and acted synergically on IL-1β stimulated IL-6 production [12]. These effects were mediated by inhibiting nuclear factor (NF)-κB signaling pathway. Indeed, we previously demonstrated that COT inhibited IL-1β induced activation of NF-κB and its translocation to the chondrocyte nucleus. Further, COT abolished IL-1β induced degradation of IκBα subunit [12].

Curcumin is the main constituent of turmeric, a yellow spice derived from the rhizomes of the plant Curcuma longa. Evidence has been published for its potency to target multiple inflammatory diseases [13]. Hydrolyzed collagen is obtained by the enzymatic hydrolysis of collagenous tissues. It is usually considered as a safe food ingredient by regulatory agencies [14, 15]. Hydrolyzed collagen contains high concentrations of glycine and proline, two amino acids essential for the stability and regeneration of cartilage [16, 17]. Green tea includes a polyphenolic fraction called epigallocatechin 3 gallate (EGCG), which exhibits anti-oxidant, anti-tumoral and anti-mutagenic activities [8].

The objectives of this study were to identify new targets of COT using genomic approaches. We compared gene expression profiles of chondrocytes treated with COT and/or with IL-1β. The proteins coded by the most important COT sensitive genes were then quantified by specific immunoassays.

## Methods

### Patients and ethics statement

Articular cartilage samples from 12 patients with knee OA (10 women and 2 men; mean age 67 years old, range 54–76 years old) were obtained at the time of total knee joint replacement surgery. All subjects provided written informed consent and the protocol was approved by ethical committee of the Catholic University of Louvain (no. B403201214793). The procedures followed were in accordance with the ethical standards of the responsible committee on human experimentation (institutional and national) and with the Helsinki Declaration of 1975, as revised in 2000.

### Chondrocytes isolation

Full-depth articular cartilage was excised and immersed in Dulbecco’s Modified Eagle Medium (DMEM) (with phenol red and 4.5 g/L glucose) supplemented with N-(2-hydroxyethyl)piperazine-N’-(2-ethanesulfonic acid) (HEPES) 10 mM, penicillin (100 U/ml) and streptomycin (0.1 mg/ml) (all from Lonza, Verviers, Belgium). After three washings, chondrocytes were released from cartilage by sequential enzymatic digestions with 0.5 mg/ml hyaluronidase type IV S (Sigma-Aldrich, Bornem, Belgium) for 30 min at 37°C, 1 mg/ml pronase E (Merck, Leuven, Belgium) for 1 h at 37°C and 0.5 mg/ml clostridial collagenase IA (Sigma-Aldrich, Bornem, Belgium) for 16 to 20 h at 37°C. The enzymatically isolated cells were then filtered through a nylon mesh (70 μm), washed three times, counted and filled to the density of 0.1 x 10^6^ cells/ml of DMEM (with phenol red and 4.5 g/L glucose) supplemented with 10% fetal bovine serum, 10 mM HEPES, 100 U/ml penicillin, 0.1 mg/ml streptomycin, 2 mM glutamine (all from Lonza, Verviers, Belgium), 20 μg/ml proline and 50 μg/ml vitamin C (Sigma-Aldrich, Bornem, Belgium) [12].

### Chondrocytes culture

Cells were seeded in a 6-well plate at the density of 0.2 x 10^6^ cells/well and cultured in monolayer for 5 days. Chondrocytes were then cultured in monolayer until confluence (for about 24 hours) in DMEM supplemented with 1% fetal bovine serum, 10 mM HEPES, 100 U/ml penicillin, 0.1 mg/ml streptomycin, 2 mM glutamine, 20 μg/ml proline and 50 μg/ml vitamin C. Only primary cultures were used to ensure the stability of chondrocyte phenotype. When human OA chondrocytes achieved confluence, the culture medium was removed and replaced by fresh culture medium with or without the combination of tested compounds (COT: 4 μg/ml curcuminoids extract + 4 μg/ml hydrolyzed collagen + 4 μg/ml green tea extract), and in the absence or in the presence of human IL-1β (10^−11^ M) (R&D System, Abingdon, UK).

Curcuminoids extract (Naturex, Avignon, France) was composed of natural extract and methylcellulose. Its content in curcuminoids was about 82% of which 75% were curcumin, 21% were demethoxycurcumin and 4% were bisdemethoxycurcumin. Hydrolyzed collagen (Gelita, Eberbach, Germany) was a mix of different peptides. In average the peptides were composed by 30 amino acids, meaning a molecular weight of about 3 kDa. Glycine and proline represented more than 35% of the total amino acids content. Green tea extract (Naturex, Avignon, France) was obtained from green tea leaves, and contained natural extract and maltodextrin. Total polyphenols content was higher than 25%, catechins content higher than 12.5% and EGCG content higher than 9.3%. Compounds were solubilized as previously described [12]. The effects of compounds were compared to controls consisting in same media without compounds and with or without IL-1β. Chondrocytes were incubated for 24 or 48 hours with the compounds and/or IL-1β. After 24 h of incubation, cells were scrapped, and ribonucleic acid (RNA) extraction was performed using RNeasy mini kit (Qiagen, Venlo, Netherlands).

After 48 h of incubation, conditioned culture media were collected for lactate dehydrogenase (LDH) release assay and then stored at -20°C until other analysis. Cells were scrapped and homogenized in 500 μl of Tris-HCl buffer by ultrasonic dissociation for 20 s at 4°C, to measure desoxyribonucleic acid (DNA) content.

### Lactate dehydrogenase release assay

Cell viability was estimated by quantifying the release of LDH in the culture supernatant as previously described [18]. A sample of the supernatant or dilutions of standard solution (LDH from rabbit muscle) was mixed with Tris buffer (10 mM Tris-HCl (pH 8.5), 0.1% bovine serum albumin) containing 800 mM lactate. Then, colorimetric reagent, 1.6 mg/ml iodonitrotetrazolium chloride (Sigma-Aldrich, Bornem, Belgium), 4 mg/ml nicotinamide adenine dinucleotide (Roche Diagnostics, Brussels, Belgium), and 0.4 mg/ml phenazine methosulfate (Sigma-Aldrich, Bornem, Belgium), was added, and the absorbance at 492 nm was read after 10 min of incubation at room temperature.

### DNA assay

DNA content was measured in the cell extracts by a fluorimetric method as described in Hoechst [19].

### RNA extraction

Total RNA was extracted using an RNeasy mini kit and reverse transcribed with SuperScript III reverse transcriptase according to the instructions of the manufacturer (Invitrogen, Merelbeke, Belgium). The yield of the extracted RNA was determined spectrophotometrically by measuring the optical density at 260 nm. The purity and quality of extracted RNA were evaluated using an Experion RNA StdSens Analysis Kit (Bio-Rad Laboratories, Hercules, CA). High-quality RNA with RNA quality indicator scores of >8 was used for microarray experiments.

### Microarray

Gene expression profiling was performed using an Illumina multisample format Human HT-12 v4 Bead-Chip. This chip contains more than 34000 probes and profiles 12 samples simultaneously on a single chip. For each sample, 250 ng of total RNA was labeled with biotin using an Illumina TotalPrep-96 RNA Amplification Kit (Ambion) according to the producer’s instructions. The biotinylated RNA probe was hybridized to the Human HT-12 v4 BeadChip. The hybridization, washing, and scanning were performed according to the manufacturer’s recommendations. The microarray images were registered and extracted automatically during the scan according to the manufacturer’s default settings. Raw expression data were acquired with GenomeStudio (Illumina) software without normalization process. The non-normalized table was then uploaded in BRB Array tools software (available at http://linus.nci.nih.gov/BRB-ArrayTools.html). Data were normalized with quantile protocol through the lumi package, included in BRB Array tools. The quantile-normalized data were filtered out based on the variance for the gene across the arrays, a feature included in BRB ArrayTools that allows the removal of unexpressed or equally expressed transcripts.

The Class Comparison Tool (BRB ArrayTools) computed the number of genes that were differentially expressed among the classes at the statistical significance level selected in the t-test menu (p-value<0.001) and created a gene list. The output gene list table was ordered by univariate p-value with the most significant genes listed first. The biologic relevance of up- and down-regulated genes was analyzed using Ingenuity Pathway Analysis (IPA) (Ingenuity Systems).

All data have been deposited in NCBI GEO and are accessible through GEO Series accession no. GSE75181 (http://www.ncbi.nlm.nih.gov/geo/query/acc.cgi?acc=GSE75181).

### Immunoassays

Immunoassays (ELISA) were used to confirm the protein production and secretion from the genes identified as being differentially expressed. Chemokine (C-X-C motif) ligand (CXCL) 6, MMP-13, bone morphogenetic protein-2 (BMP-2), stanniocalcin 1 (STC1) and serpin E1 productions were measured by specific enzyme amplified sensitivity immunoassays (RnD Systems, Abingdon, United Kingdom). CXCL6, MMP-13, BMP-2, STC1 and serpin E1 productions were measured in culture supernatants.

### Statistical analysis

The class comparison between groups of Arrays Tool computed a t-test separately for each gene. Since data from the control (ctrl), COT, IL-1β and COT IL-1β conditions came from the same patient, we used the option “paired t-test”, available in BRB ArrayTools, to improve the statistical power of the analysis. We used a paired t-test p-value threshold of less than 0.001 in our analysis.

Immunoassays results were normalized to the DNA content of the cells and expressed in the text as the mean ± standard error mean (SEM). Statistical significance is assessed using a Student’s t-test, performed with Graph Pad Prism software, version 6. Differences were considered statistically significant at p-value < 0.05.

## Results

### Global microarray results

From among 34602 probes, 4168 were filtered out. Differential analysis was done from these 4168 filtered probes. The class comparison test was made between IL-1β and ctrl conditions, between COT IL-1β and IL-1β conditions and between COT and ctrl conditions. The class comparison test was based on a paired t-test where ctrl, COT, IL-1β and COT IL-1β conditions were paired for each patient (n = 12). Probes with a p-value less than 0.001 were chosen as up- or down-regulated ones. 2549 genes were differentially expressed between IL-1β and ctrl conditions, 2280 genes were differentially expressed between COT IL-1β and IL-1β conditions and 1907 genes were differentially expressed between COT and ctrl conditions. The cut-off ratio (IL-1β/ctrl, COT IL-1β/IL-1β and COT/ctrl) that we used to consider a gene differentially expressed between two conditions was 2 for an up-regulated gene and 0.5 for a down-regulated gene. In order to facilitate the comparison, for down-regulated genes, we expressed data with the inverse of the ratio 0.5, which is -2. These values correspond to fold change of expression. The class comparison test between IL-1β and ctrl showed 552 up-regulated probes and 297 down-regulated probes. The class comparison test between COT IL-1β and IL-1β showed 424 up-regulated probes and 577 down-regulated probes. Finally, the class comparison test between COT and ctrl showed 323 up-regulated probes and 259 down-regulated probes.

### Main pathways identified

Based on the standard networks generated by IPA, the analysis was deepened in order to identify specific OA pathways. A significant number of genes that were differentially expressed between ctrl, COT, IL-1β and COT IL-1β conditions were categorized as belonging to inflammation, cartilage metabolism and angiogenesis key regulated pathways. Ratios IL-1β/ctrl, COT IL-1β/IL-1β and COT/ctrl of >2 and <-2 were considered relevant. The 3 key pathways were analyzed in detail.

### Inflammation network

Hundreds of inflammatory mediators were demonstrated to be up-regulated in IL-1β compared to ctrl condition. Those with the highest fold change of expression were presented in Table 1. Most of these mediators were then showed to be down-regulated in COT IL-1β compared to IL-1β condition. These mediators belonged to different categories: inflammatory cytokines, chemokines, enzymes, and their related partners. Only four inflammatory mediators were differentially expressed between COT and ctrl conditions.

**Table 1:** Genes differentially expressed between IL-1β and ctrl, COT IL-1β and IL-1β, COT and ctrl conditions.

	IL-1β/ctrl		COT IL-1β/IL-1β		COT/ctrl	
Pathway, type of compound	Up-regulated	Down-regulated	Up-regulated	Down-regulated	Up-regulated	Down-regulated
Inflammation						
Cytokines	IL8 (126.58); IL6 (109.89); TNFAIP6 (29.41); IFI44L (8.33)	IL11RA (-3.2); CYTL1 (-3.12)		C1QTNF1 (-17.54); IL6 (-13.7); IFIT1 (-13.7); TNFAIP6 (-5.56); IL8 (-3.13)		C1QTNF1 (-1.45)
Chemokines	CXCL6 (104.17); CCL20 (100); CXCL1 (83.33); CCL8 (47.62); CXCL2 (33.33); CCL5 (33.33); CXCL5 (17.86); CXCL10 (10.2); CCL2 (10)			CXCL6 (-52.63); CCL8 (-31.25); CXCL5 (-12.66); CXCL1 (-10.20); CXCL2 (-10); CCL5 (-8.33); CXCL10 (-8.33); CCL3L3 (-5.26); CCL2 (-5.26); CCL3 (-4.55); CCL3L1 (-3.13); CCL20 (-2.94); CCL7 (-2.86); CCL13 (-2.38)		CCL2 (-2.5)
Enzymes	NOS2A (50); SOD2 (31.25); PTGS2 (31.25); PTGES (19.61)	GPX3 (-4.47); DDAH1 (-4.16); GSTM2 (-3.53); GSTM1 (-3.24)	GPX3 (2.89)	NOS2A (-24.39); PTGES (-14.08); PTGS2 (-5.56)	PTGS2 (2.77)	
Other	LCN2 (16.67); IER3 (16.13); NFKBIA (11.49); CFB (10.87); NFKBIZ (8.33)		ZFP36 (4.08); ULBP1 (3.7); SNIP1 (3.54); CD83 (2.33)		ULBP1 (4.56)	
Anabolism	BMP2 (8.33); BMP6 (4)	CTGF (-13.62); GREM1 (-7.62); FGFR3 (-3.02); BMP4 (-2.76); CILP (-2.12)	CTGF (3.54)	FGF2 (-5.56); BMP2 (-3.85); BMP6 (-3.45)	GDF15 (8.07)	IGFBP5 (-4.35)
Catabolism	MMP13 (23.26); MMP1 (9.09); CTSS (2.33)	ADAMTS1 (-3.91)		MMP13 (-12.82); MMP1 (-3.33); ADAMTS5 (-3.23); ADAMTS9 (-3.03)		ADAMTS5 (-4.00); ADAMTS1 (-3.23)
Angiogenesis	ECGF1 (7.69); RCAN1 (4.17); STC1 (3.33); BDKRB1 (3.03); AQP9 (2.86); HBEGF (2.04)	ANGPTL2 (-5.94); VEGFB (-2.51)	SERPIN E1 (4.28); SERPIN C1 (2.45); HBEGF (2.05)	ECGF1 (-7.14); VCAM1 (-3.85); STC1 (-3.7); TFPI2 (-3.45); FBLN5 (-2.94); BDKRB1 (-2.7); PAFAH1B1 (-2.13); VEGFC (-2.08); SERPIN A1 (-2.08)	HBEGF (4); SERPIN E1 (3.1); SERPIN C1 (2.52); RCAN1 (2.51)	ANGPTL2 (-4.17); VCAM1 (-4); BMPER (-2.08)

Values in parentheses correspond to fold change of expression.

The most regulated chemokine was CXCL6. This gene was strongly up-regulated by IL-1β (104.17-fold, p<10^−7^, FDR<10^−7^) and the IL-1β stimulated CXCL6 gene expression was strongly down-regulated by COT (-52.63-fold, p<10^−7^, FDR<10^−7^).

**Fig 1:**
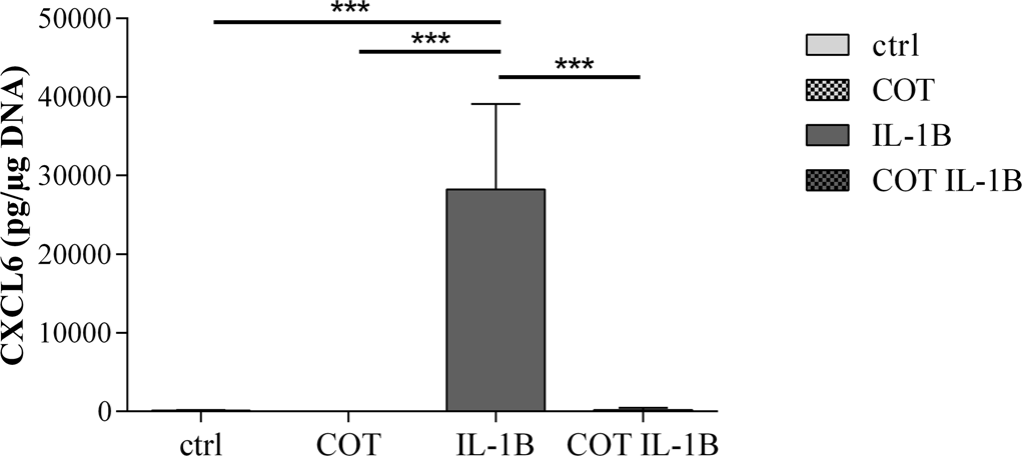
CXCL6 production by human chondrocytes in ctrl, COT, IL-1β and COT IL-1β conditions. Results were expressed as mean ± 95% confidence intervals of 12 independent experiments performed with chondrocytes isolated from 12 different patients. ***p-value<0.001. CXCL6 = chemokine (C-X-C motif) ligand 6.

To validate the differential expression of CXCL6 by OA human chondrocytes, we measured CXCL6 protein in the culture medium by immunoassay. As illustrated in Fig 1, the level of CXCL6 protein production was significantly increased by IL-1β (ctrl: 86.2 ± 52.9 pg/μg DNA, IL-1β: 28245.4 ± 4934.7 pg/μg DNA, p<0.001) and the IL-1β stimulated CXCL6 protein production was significantly decreased by COT (185.9 ± 114.6 pg/μg DNA, p<0.001) (Fig 1).

### Cartilage metabolism pathway

The comparison between IL-1β and ctrl conditions, and between COT IL-1β and IL-1β conditions highlighted several factors involved in both cartilage anabolism and catabolism (Table 1). The most IL-1β up-regulated gene in the catabolic pattern was MMP-13 (23.26-fold, p<10^−7^, FDR<10^−7^) and in the anabolism pattern BMP-2 (8.33-fold, p<10^−7^, FDR<10^−7^). Both IL-1β stimulated MMP-13 and BMP-2 genes expression were down-regulated by COT (-12.82-fold, p<10^−7^, FDR<10^−7^ and -3.85-fold, p = 8.79x10^-5^, FDR = 0.00024, respectively).

**Fig 2:**
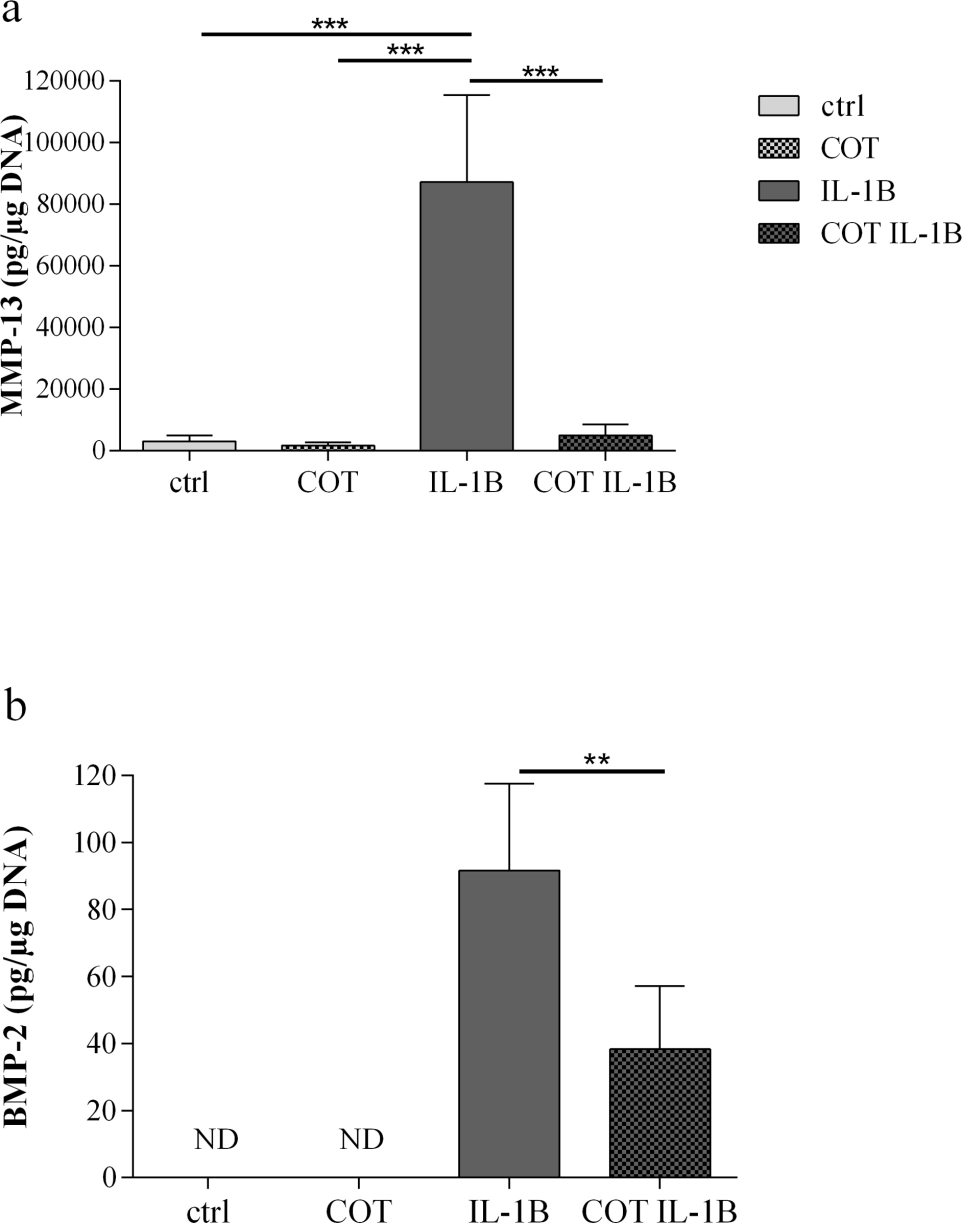
MMP-13 and BMP-2 productions by human chondrocytes in ctrl, COT, IL-1β and COT IL-1β conditions. Results were expressed as mean ± 95% confidence intervals of 12 independent experiments performed with chondrocytes isolated from 12 different patients. **p-value<0.01, ***p-value<0.001. (a) MMP-13 = matrix metalloproteinase-13, (b) BMP-2 = bone morphogenetic protein-2, ND = non detectable.

These observations were confirmed at the protein level. MMP-13 production was significantly increased by IL-1β (ctrl: 3033.8 ± 875.2 pg/μg DNA, IL-1β: 87115.8 ± 12806.2 pg/μg DNA, p<0.001). This IL-1β stimulating effect was strongly inhibited by COT (4910 ± 1621.6 pg/μg DNA, p<0.001) (Fig 2A). The level of BMP-2 protein production was undetectable in basal conditions, reached 91.6 ± 11.8 pg/μg DNA in the presence of IL-1β. In the presence of COT, the IL-1β stimulated BMP-2 protein production significantly decreased to 38.3 ± 8.6 pg/μg DNA (p = 0.001) (Fig 2B).

### Angiogenesis pathway

As revealed by the network analysis, the angiogenesis pathway was differentially activated between ctrl, COT, IL-1β and COT IL-1β conditions (Table 1). STC1 gene expression was strongly up-regulated by IL-1β (3.33-fold, p = 3x10^-7^, FDR = 1.12x10^-6^). This IL-1β effect was down-regulated by COT (-3.7-fold, p = 4.1x10^-6^, FDR = 2.43x10^-5^). Serpin E1 gene expression was not affected by IL-1β compared to ctrl while Serpin E1 gene expression was up-regulated in COT IL-1β condition, compared to IL-1β condition (4.28-fold, p = 2.25x10^-5^, FDR = 8.3x10^-5^). Moreover, serpin E1 gene expression was up-regulated by COT in ctrl condition (3.1-fold, p = 1.2x10^-6^, FDR = 1.27x10^-5^).

**Fig 3:**
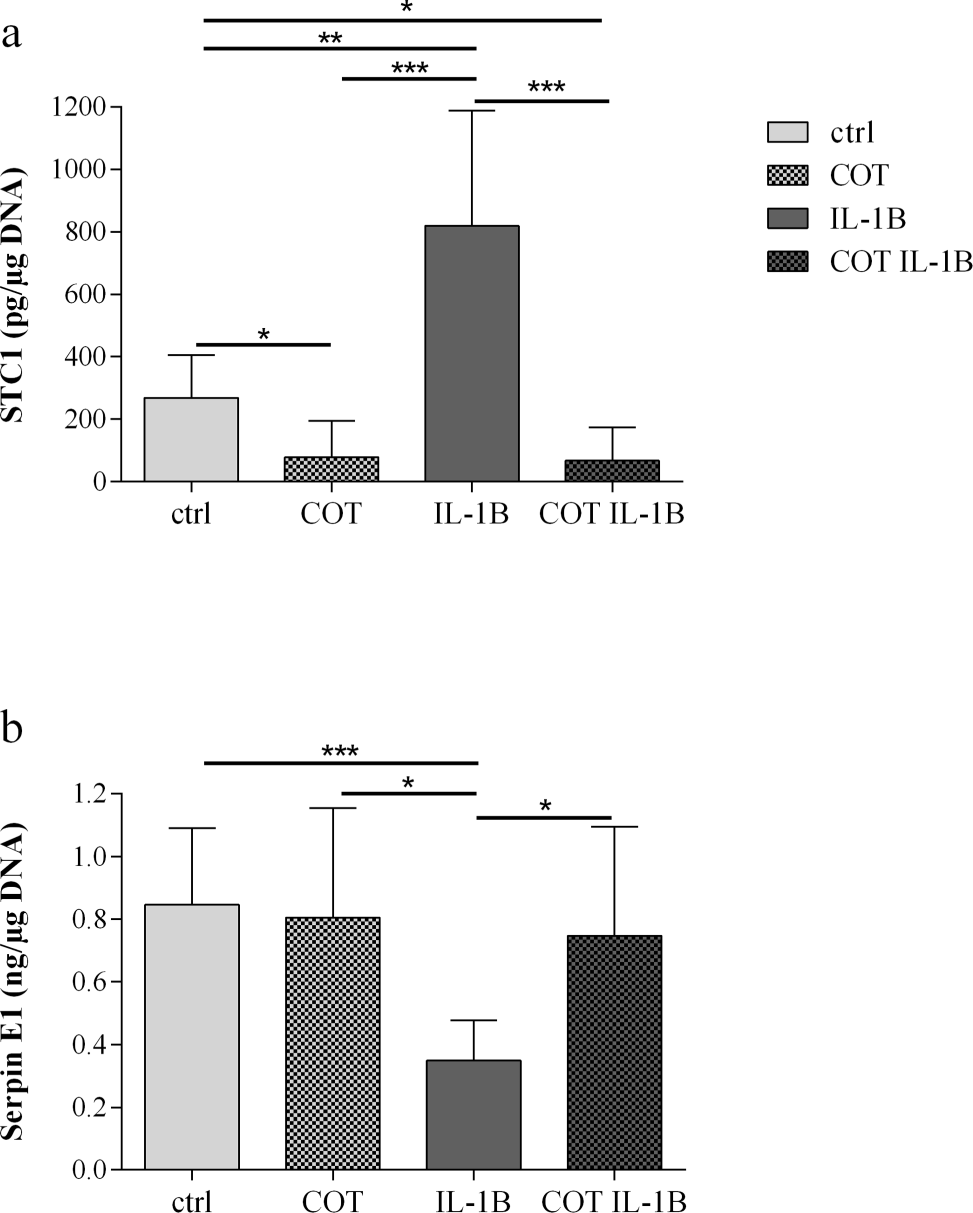
STC1 and serpin E1 productions by human chondrocytes in ctrl, COT, IL-1β and COT IL-1β conditions. Results were expressed as mean ± 95% confidence intervals of 12 independent experiments performed with chondrocytes isolated from 12 different patients. *p-value<0.05, **p-value<0.01, ***p-value<0.001. (a) STC1 = stanniocalcin 1, (b) serpin E1

Consistent with the microarray data, a significant increase of STC1 production was induced by IL-1β (ctrl: 268.6 ± 62.3 pg/μg DNA, IL-1β: 819.3 ± 167.4 pg/μg DNA, p = 0.005). This IL-1β stimulating effect was significantly decreased by COT (67.7 ± 47.9 pg/μg DNA, p<0.001) (Fig 3A). Moreover, it was shown that COT significantly decreased STC1 production in basal condition (78.8 ± 52.8 pg/μg DNA, p = 0.030) (Fig 3A). Serpin E1 production was down-regulated by IL-1β (ctrl: 0.85 ± 0.11 ng/μg DNA, IL-1β: 0.35 ± 0.06 ng/μg DNA, p<0.001). COT fully reversed the inhibitory effect of IL-1β (0.75 ± 0.16 ng/μg DNA, p = 0.028) (Fig 3B).

## Discussion

Transcriptome analysis appears to be a promising approach in the understanding of complex disorders such as OA. Enhanced implementation of functional genomics is needed to substantially augment translation to treatment development and disease management [20]. Functional studies have so far revealed that effects on gene expression are likely to be one of the main mechanisms through which OA susceptibility is acting. Genetic, transcriptomic and epigenetic data will allow us to use the genetic discoveries for informed development of new OA biological treatments [21].

Chondrocytes play a central role in osteoarthritis. Indeed, a single session of noninvasive loading leads to the development of OA-like morphological and cellular alterations in articular cartilage [22]. For example, OA chondrocytes under conditions of compression upregulated Runx-1, the hematopoietic lineage determining transcription factor [23]. Mechanical loading stimulated the expression and release of nerve growth factor (NGF) by chondrocytes. This may mediate OA pain [24]. Mechanical compression can also activate Smad2/3P signaling by chondrocytes [25]. Human primary chondrocytes were treated with IL-1β to mimic OA chondrocyte metabolic responses [12]. Our microarray analysis demonstrated that numerous genes involved in the inflammation, cartilage anabolism/catabolism, and angiogenesis key pathways were differentially expressed between IL-1β and ctrl or between COT IL-1β and IL-1β or between COT and ctrl conditions.

Concerning the inflammation pathway, we confirmed the stimulating effect of IL-1β on a large number of cytokine, chemokine and enzyme genes, mainly IL-8, IL-6, CXCL6, tumor necrosis factor alpha induced protein (TNFAIP)6, interferon-induced protein 44-like (IFI44L), chemokine (C-C motif) ligand (CCL)20, CXCL1, CCL8, CXCL2, CCL5, CXCL5, CXCL10, CCL2, nitric oxide synthase 2, inducible (NOS2A), superoxide dismutase (SOD)2, prostaglandin-endoperoxide synthase (PTGS)2 and prostaglandin E synthase (PTGES). Interestingly, COT significantly down-regulated the expression of these key genes in the inflammatory pathway. The most up-regulated chemokine in IL-1β condition was CXCL6. This finding is consistent with those of a previous studies showing that CXCL6 expression was increased in fibroblasts and in human chondrocytes after stimulation with IL-1β [26, 27]. It was shown that CXCL6 was overexpressed in synoviocytes derived from patients with OA [26]. CXCL6 gene expression was also higher in hip OA cartilage than in normal cartilage [28]. CXCL6 intervened in neutrophils attraction and was therefore implicated in inflammatory process [29, 30]. This is also on this gene that COT had its strongest inhibitory effect. However, recently, it was shown that CXCL6 was found in extracellular matrix of healthy cartilage, bound with proteoglycans [31]. The inhibition of CXCL6 is a major effect of COT which support its use in inflammatory condition. More surprisingly was the upregulation of PTGS2 with COT compared to controls. However, the fold change of expression was 2.77, which is relatively low compared to the fold change of expression obtained with IL-1β compared to controls (31.25). Moreover, PTGS2 was down-regulated with COT IL-1β compared to IL-1β (-5.56). We have already shown that COT reduced PTGS2 expression by chondrocytes stimulated by IL-1β but had no effect in basal conditions [12].

The degradation of cartilage is a key process in OA. Mainly MMP-13 plays a key role in this process. This MMP was shown to be up-regulated in OA cartilage and was presented as the most potent MMP involved in type II collagen degradation [32–34]. Interestingly, COT drastically inhibited IL-1β stimulated MMP-13 gene expression and protein production. This indicates that COT could slow down cartilage degradation in OA through the inhibition of MMP-13. Even if BMP-2 is classically implicated in cartilage and bone repair, our results were congruent with those of a study indicating that IL-1β increased BMP-2 expression in human chondrocytes [27]. BMP-2 expression was significantly greater in OA cartilage than in normal cartilage. Patients with severe X-ray knee OA showed significantly increased BMP-2 levels in the serum and synovial fluid compared with those with moderate OA [35]. BMP-2 concentrations in the serum and synovial fluid of knee OA patients were closely related to the radiographic and symptomatic severity of knee OA [35]. Moreover, an increase in BMP-2 levels in mice resulted in severe aggravation of osteophyte formation [36]. Interestingly, COT decreased IL-1β stimulating effect on BMP-2 gene expression and protein production. This can be interpreted as a beneficial effect since the concentration of BMP-2 is correlated with the severity of the disease.

The importance of angiogenesis in OA is now well known. Blood vessels have been observed in OA cartilage and an overexpression of pro-angiogenic factors by OA chondrocytes was demonstrated [37–39]. Data generated in the present microarray showed that various key mediators of angiogenesis were modulated by IL-1β and COT. More particularly, we observed an up-regulation of STC1 gene in IL-1β condition and an inhibition of this effect by COT. A study recently showed that STC1 was up-regulated, at both the gene and protein levels, in inflamed area compared with normal/reactive area of OA synovial membrane [40]. STC1 plays roles in angiogenesis via the vascular endothelial growth factor (VEGF)/VEGF receptor 2 pathway [41–43]. This indicated that COT could protect OA cartilage against blood vessels invasion by down-regulating key pro-angiogenic mediators. Finally, we have focused our attention on serpin E1 gene. This gene was up-regulated in COT and COT IL-1β conditions, compared with ctrl and IL-1β conditions, respectively. Serpin E1 is also known as plasminogen activator inhibitor-1 (PAI-1). It has been described as a member of the serine protease inhibitor superfamily that inhibits the activation of both plasminogen activator and urokinase-type plasminogen activator, which act in fibrinolysis [44]. PAI-1 regulates angiogenesis via effects on extracellular matrix proteolysis and cell adhesion. PAI-1 inhibited VEGF/VEGF receptor 2 signaling [45]. Given that COT increased PAI-1 synthesis, COT could inhibited VEGF pathway and therefore angiogenesis.

We are aware that a larger IPA analysis could probably add more information. However, we decided to focus our analysis on these pathways for the moment. One limitation of our study is that chondrocytes were treated with 4 μg/ml COT, which is a high dose. Additional studies are needed to evaluate the amount of COT that would reach chondrocytes if COT was ingested per os. Indeed, there is a lack of information about dietary supplements bioavailability. Particularly, natural curcumin is known for its very low bioavailability. For example, the average peak serum concentrations after oral intake of 4, 6 and 8 g of curcumin per day were 0.51 ± 0.11, 0.63 ± 0.06 and 1.77 ± 1.87 μM, respectively [46]. The serum concentration of curcumin peaked at 1–2 h after oral intake of curcumin and gradually declined within 12 hours. Urinary excretion of curcumin was undetectable [46]. This mean that the concentrations of curcuminoids extract tested in this *in vitro* study (4 μg/ml _~_ 10 μM) are superior to those found in plasma after oral administration of high doses of natural curcumin. Therefore, the extrapolation of our *in vitro* data to human nutrition must be done with caution. However, many effort have been made to increase curcumin bioavaibility. This can be made by including curcumin in a complex of phospholipids for example. Recently, we performed a Phase I pharmacokinetics study on Flexofytol, a high bioavailable turmeric extract with a water solubility increased 4000 times, that was run on 2 groups of 12 healthy individuals. Each group received orally 1 (42 mg curcumin) or 2 capsules (84 mg of curcumin) of Flexofytol respectively. With 2 capsules administered orally, the mean of Cmax on 12 individuals was 0.9 μM, with a statistical extrapolation at 1.6 μM with 4 capsules (administering 84 mg and 168 mg of curcumin respectively). These values are closer with those used in our *in vitro* study.

## Conclusion

Our microarray analysis has revealed that thousands of genes were sensitive to COT in human chondrocytes. These genes are associated with important pathophysiologic processes in OA: inflammation, anabolism/catabolism and angiogenesis. They represent key targets for OA treatment. This paper helps to understand how the COT mixture acted on OA pathogenesis. As expected, we confirmed that the COT mixture acted on inflammation and cartilage metabolism. Additionally, we provided new information regarding the action of COT on angiogenesis key pathway. These findings give a supplementary scientific rationale for the use of these natural ingredients in the management of OA.
